# The Effects of Intense Physical Activity on Stress in Adolescents: Findings from Korea Youth Risk Behavior Web-Based Survey (2015–2017)

**DOI:** 10.3390/ijerph16101870

**Published:** 2019-05-27

**Authors:** Hwi Jun Kim, So Yeon Oh, Doo Woong Lee, Junhyun Kwon, Eun-Cheol Park

**Affiliations:** 1 Department of Public Health, Graduate School, Yonsei University, Seoul 03722, Korea; kimhwijun88@yuhs.ac (H.J.K.); sarahoh@yuhs.ac (S.Y.O.); doowoonglee@yuhs.ac (D.W.L.); judekwon@yuhs.ac (J.K.); 2 Institute of Health Services Research, Yonsei University, Seoul 03722, Korea; 3 Department of Preventive Medicine, Yonsei University College of Medicine, Seoul 03722, Korea

**Keywords:** intense physical activity, stress, residence type, suicidal ideation, Korea Youth Risk Behavior Web-Based Survey

## Abstract

The purpose of this study was to investigate the association between intense physical activity and stress in Korean adolescents. The study used data from the Korea Youth Risk Behavior Web-Based Survey (KYRBWS), 2015–2017, that included 170,359 responses from Korean adolescents. Intense activity and stress were measured by self-diagnosis. Additionally, the chi-square test and multiple logistic regression analyses were used. It was revealed that 78.9% of Korean adolescents were exposed to stress. Students who engaged in physical activity more than five times per week were less likely to be stressed than those who did not (boys odds ratio (OR): 0.79, confidence interval (CI): 0.78–0.80, *p* for trend: <0.0001; girls OR: 0.77, CI: 0.75–0.79, *p* for trend: <0.0001). The results indicated the same tendency among both boys and girls. The results of subgroup analysis revealed that students living with relatives or in childcare facilities were more likely to experience stress if they had insufficient exercise. In addition, the results confirmed that the probability of suicidal ideation increased as the frequency of exercise decreased. This study suggests that intense physical activity in Korean adolescents has a positive effect on stress management in both boys and girls. Hence, physical activity should be encouraged and implemented for managing stress.

## 1. Introduction

Stress is an organism’s response to a stressor such as an environmental condition [1,2]. This can affect the body physically or psychologically. A Canadian expert has categorized stress into good stress and bad stress and defined these as eustress and distress, respectively. Although eustress may manifest as an immediate burden, it is a stress to develop life in the future. Distress has been defined as being able to cause anxiety, depression, and other symptoms that persist despite coping or adaptation [3,4,5]. Many previous studies that we refer to suggested that stress is related to mental health, such as depression and suicide, in terms of distress, and may also affect sleep, cardiovascular disease, and immune system disorders.

Adolescence represents the transition from childhood to adulthood and is a period characterized by physical, physiological, psychological, and mental maturation; thus, this period is marked by an unstable psychological state [6,7,8]. An unstable psychological state is caused by social factors such as schooling, personal appearance, reason and friendship, and career-related concerns; these factors can lead to stress and negatively affect the psychological state of adolescents [9,10]. 

According to the “Youth Statistics” data published by the Ministry of Health and Welfare in 2007, the perceived stress level in Korean adolescents decreased from 49.9% in 2007 to 39.8% in 2017, but 4 out of 10 Korean adolescents still experience stress [11]. Therefore, continuous efforts to improve these phenomena are needed.

As a way to manage stress, governments and professional organizations worldwide offer various measures, such as psychotherapy, exercise therapy, and mental–social interventions [12,13,14,15,16,17]. The Korea Centers for Disease Control and Prevention suggest that exercise is effective in managing stress. Further, exercise is effective in improving personal health, as well as maintaining interpersonal relationships [18,19]. According to the results of the Korea Sports Promotion Foundation, it is suggested that about 10–20 min of intense physical activity can help manage mental health [20]. A number of previous studies have also provided supporting evidence [21,22]. However, few studies have investigated the relationship between vigorous physical activity and stress. Therefore, we focused on determining how intense physical activity might be related to stress.

## 2. Materials and Methods

### 2.1. Study Participants

We obtained data for our study population from the 2015–2017 Korea Youth Risk Behavior Web-Based Survey (KYRBWS), a cross-sectional, nationwide survey conducted by the Korea Centers for Disease Control and Prevention (KCDC) (approval number 117058). The total number of survey participants during the three years of the study was 195,847. In the database, people who responded to “how many days included over 20 min of intense physical activity during the last 7 days” were surveyed from 0 to 7 days, and non-responders were excluded from the study. Examples of vigorous physical activity in this survey included jogging, soccer, basketball, climbing, cycling, and swimming. Further, we excluded data missing from the time spent sitting and studying on weekdays (*n* = 25,488). In addition, we included the samples that had responses to questions regarding gender, age, household income, residence type, number of days of weight training per week, time spent sitting and studying on weekdays, grade, alcohol consumption, smoking, depression, and suicidal thoughts. Finally, 170,359 samples (90,041 boys and 80,418 girls) were included as representative of Korean adolescents. 

### 2.2. Variables

The number of days of intense physical activity per week was the main dependent variable in this study. In the KYRBWS, the frequency of weekly intense physical activity was reported by respondents using the responses “no”, “1 day”, “2 days”, “3 days”, “4 days”, “5 days”, “6 days” or “7 days” per week.

The independent variable was stress. The questionnaire asked “how often do you feel stress?”. Possible answers included “feel very much”, “feel a lot”, “feel a little”, “not feel much”, “not feel at all”. We classified people who responded ”feel very much” and “feel a lot” into a stress group, and classified the remaining respondents into a non-stress group. Furthermore, several covariates including sociodemographic, economic, and health-related characteristics were assessed. Sociodemographic characteristics included ages of 14 (middle school, grade 1), 15 (middle school, grade 2), 16 (middle school, grade 3), 17 (high school, grade 1), 18 (high school, grade 2), and 19 (high school, grade 3). Economic characteristics included household income (low, medium-low, medium, medium-high, high), residence type (live with family, live with relatives, live in accommodation, in a dormitory, live in a childcare facility), and grade (high, medium-high, medium, medium-low, low). Health-related characteristics included the number of days of weight training per week (non-weight training, more than 5 days, 4 days, 3 days, 2 days, or 1 day per week), alcohol consumption, smoking, depression, suicidal thoughts, and time spent sitting and studying on weekdays (<8, 8 ≤ *n* < 12, 12 ≤ *n* < 16, 16 ≤ *n* < 20).

### 2.3. Statistical Analysis

The chi-square test and multiple logistic regression analysis were used to analyze the data. The chi-square test was used to examine the significant difference in stress depending on intense physical activity. Multiple logistic regression analysis was used to determine odds ratios (ORs) and 95% confidence intervals (CIs) after adjusting for covariates. In these subgroup analyses, we used the Cochran–Armitage Test to explore the two-sided *p*-value for trend in each of the variables and stress to relate the number of days of intense physical activity per week with stress. For all data analysis, we used SAS version 9.4 (SAS Institute, Cary, NC, USA) and the significance level was set at *p*-value < 0.05, as well as at *p*-value for trend <0.05. 

## 3. Results

### 3.1. Study Participants

Table 1 presents the general characteristics of study participants who analyzed the stress of boys and girls by socio-demographic factors, socio-economic status, health behavior, and other related variables (number of days of weight training, time spent sitting and studying on a weekday, grade). In the chi-square test of stress and all covariates, the *p*-value was <0.0001, which was significant. In this analysis, 73.2% of boys and 84.5% of girls responded that they felt stressed. We observed that girls had a lower frequency of exercise than boys. In addition, we found that the stress response rate tended to decrease with groups that exercise frequently for both boys and girls.

### 3.2. Factors Associated with Physical Activity and Stress

Using logistic regression analysis, Table 2 demonstrates the relationship between stress and the number of days of intense physical activity per week. Regarding the number of days of intense physical activity per week, groups with frequent physical activity were observed to have a tendency towards a reduced stressed odds ratio, which was significant. However, boys who engaged in physical activity one to two times per week were found to have similar levels of stress to the non-physical activity group. In addition, adolescents tended to feel more stressed as the amount of time spent sitting and studying on a weekday increased. Furthermore, the analysis found that as grades became poorer, more stress was experienced. We verified the significance of linear trends for each analysis through the *p* for trend analysis. For both boys and girls, the *p* value for trend was <0.0001, which was significant.

### 3.3. Association between Intense Physical Activity and Stress Stratified by Household Income, Residence Type, Time Spent Sitting and Studying on a Weekday, Grade, Depression, and Suicidal Ideation

Table 3 illustrates the results of subgroup analysis associating stress with intense physical activity and household income, type of residence, time spent sitting and studying on a weekday, grades, depression, and suicidal ideation. In this analysis, when the students who do not exercise even once per week are taken as the criteria, the more intense physical activity is performed in most variables, the less likely they are to be stressed. 

In most subgroup analyses, frequent intense physical activity was associated with reduced stress. In particular, the analysis of girls’ residence type showed that frequent intense physical activity was found to be effective for girls who lived with relatives and those who lived in childcare facility groups. Furthermore, the analysis of depression and suicidal ideation also showed that boys and girls were more likely to participate in physical activity and experience fewer mental health problems.

## 4. Discussion

Our study was conducted to investigate the relationship between intense physical activity and stress in Korean adolescents. Our results indicated that about 78.9% of Korean youth felt stressed. We were able to confirm that the response rate of girls feeling stressed was higher than boys. However, both boys and girls were able to confirm that when they engaged in more intense physical activity, their response rate to stress was lower.

Some argue that the right amount of stress can further improve the lives of adults [23]. However, adolescence is subject to sudden physical changes and is vulnerable to various external factors; sustained stress during adolescence may have a negative impact, both physically and psychologically [24,25]. Therefore, in order for adolescents to overcome stress, it is necessary to understand the causes of stress and learn how to cope with them. There are numerous factors that cause stress in our lives, but there are also many ways to manage this stress [26,27,28,29]. Experts suggest that sleep therapy, diet, mental training, and physical activity are efficient ways to manage stress. In line with our research findings, according to a study involving 7–8 year old girls in the U.S. conducted by the American Psychological Association, exercise is effective in stress management [30]. The positive effects of exercise on stress have been proven both in quantitative and qualitative studies [31,32]. Studies conducted in Thailand showed that the discharge of cortisol and epinephrine, which are stress-inducing hormones, was decreased for 24 h by jogging five times per week [33,34,35]. 

Through this study, we were able to identify the trends associated with stress in Korean adolescents. First, the stress experienced by Korean students should no longer be regarded as an issue for students to manage themselves. In our study, 78.9% of Korean students answered that they are under stress. Of all the respondents, 73.2% of boys and 84.5% of girls answered that they are under stress. This means that a large number of students are exposed to factors that cause stress. Further, stress causes physical, psychological, and mental diseases. Our study also found that stress was related to depression or suicidal ideation. Therefore, we should not demand that adolescents address their stress themselves. This suggests that governments, academia, schools, and families need to take measures to effectively manage stress. Second, regular intense physical activity may be effective in adolescent stress management. Students, both boys and girls, who engaged in intense physical activity for three days or more each week were less stressed than those who did not. However, the stress experienced by people who exercise less than twice per week is similar to those who do not exercise at all. This may mean that a certain frequency of physical activity causes a physiological mechanism to reduce stress. Despite the apparent effectiveness of physical activity, some students may not be able to engage in physical activity due to a lack of time. Therefore, we propose that schools guarantee that physical education is provided at least three times per week for the effective stress management of Korean adolescents. Third, girls are more vulnerable to stress than boys. About 73% of the boys responded that they felt stressed, compared to about 85% of the girls. It is also possible that girls are more stressed than boys because of cultural expectations and gender-stereotypical behaviors [17]. However, considering the girls’ stress response rate is 85%, it is need a stress management system for girls. Therefore, as an alternative, we believe that developing an exercise program specifically for girls can help to effectively manage their stress. These observations are in line with the guidelines of the World Health Organization and American College of Sports Medicine [36,37]. Therefore, while it is best to engage in intense physical activity as much as possible, when considering busy students’ schedules, it is necessary to examine ways to institutionalize physical activity so that students can exercise at least three times per week.

However, our study has some limitations. First of all, since this was a cross-sectional study, a causal relationship between intense physical activity and stress could not be established. Second, the KYRBWS questions investigate the average of the past year. The data used in this study were mostly based on self-reported surveys; hence, questions on socioeconomic status, health behavior, and the intense physical activity covariate may have been subjected to recall bias. Third, the measurement of stress depends on self-diagnosis, and qualitative research through experimental testing is necessary for the accurate measurement of stress. Fourth, the effect of intense physical activity on stress was obvious. However, the effect was not significant. The stress experienced by students who engaged in intense physical activity for three days did not differ greatly from those who engaged in intense physical activity for five days or more. Nevertheless, students with no physical activity, once-weekly intense physical activity, or twice-weekly intense physical activity were about 1.3 times more stressed. 

Despite these limitations, our research has several strengths. First, this study used primary data suitable for the study of Korean adolescents. These data are sufficient to represent the phenomenon for Korean adolescents, since 170,000 respondents were included. Second, we have accumulated six years of data to study the phenomenon that occurs in adolescents. Therefore, the correlation between stress and intense physical activity in adolescents is reliable. Third, it provides information on the appropriate level of intense physical activity required for Korean adolescents to manage stress. Our study showed that intense physical activity in each assay was not significantly different from more than five days per week when it was more than three times per week. However, students who engaged in intense physical activity less than two times per week were more stressed. Therefore, when establishing a system and policy for the mental health management of adolescents, the results of this study could be utilized; intense physical activity three times per week could be made mandatory in class or club activities.

## 5. Conclusions

Our study identified that intense physical activity in Korean adolescents is associated with a decrease in stress. In addition, the least amount of stress was found in adolescents who engaged in frequent intense physical activity. Therefore, we propose to establish a system to guarantee physical activity time for the health of Korean adolescents. If intense physical activity is difficult to perform on a daily basis, we suggest engaging in intense physical activity at least three times per week.

## Figures and Tables

**Table 1:** General characteristics of study observations (2015–2017).

Variable	Boys						*p*-Value	Girls						*p*-Value
	Total		Stress		Non-Stress			Total		Stress		Non-Stress		
	*n*	(%)	*n*	(%)	*n*	(%)		*n*	(%)	*n*	(%)	*n*	(%)	
**Number of days of intense physical activity per week**							<0.0001							<0.0001
More than 5 days a week	21,876	24.3	15,161	69.3	6715	30.7		7065	8.8	5779	81.8	1286	18.2	
4 days a week	8423	9.4	6043	71.7	2380	28.3		4004	5.0	3291	82.2	713	17.8	
3 days a week	16,197	18.0	11,739	72.5	4458	27.5		10,182	12.7	8370	82.2	1812	17.8	
2 days a week	17,154	19.1	12,954	75.5	4200	24.5		14,471	18.0	12,161	84.0	2310	16.0	
1 days a week	14,123	15.7	10,749	76.1	3374	23.9		18,610	23.2	15,814	85.0	2796	15.0	
Non-physical activity	12,268	13.6	9302	75.8	2966	24.2		25,986	32.4	22,418	86.3	3568	13.7	
**Age**							<0.0001							<0.0001
14 (middle school 1)	15,558	17.3	10,420	67.0	5138	33.0		14,266	17.8	11,221	78.7	3045	21.3	
15 (middle school 2)	16,044	17.8	11,385	71.0	4659	29.0		14,443	18.0	11,888	82.3	2555	17.7	
16 (middle school 3)	16,420	18.2	11,823	72.0	4597	28.0		15,153	18.9	12,516	82.6	2637	17.4	
17 (high school 1)	14,356	15.9	10,755	74.9	3601	25.1		12,644	15.7	10,948	86.6	1696	13.4	
18 (high school 2)	14,814	16.5	11,581	78.2	3233	21.8		12,709	15.8	11,335	89.2	1374	10.8	
19 (high school 3)	12,849	14.3	9984	77.7	2865	22.3		11,103	13.8	9925	89.4	1178	10.6	
**Household income**							<0.0001							<0.0001
High	10,349	11.5	6579	63.6	3770	36.4		5888	7.3	4325	73.5	1563	26.6	
Medium-high	25,118	27.9	17,845	71.0	7273	29.0		20,807	25.9	16,997	81.7	3810	18.3	
Medium	40,329	44.8	29,882	74.1	10,447	25.9		40,262	50.1	34,445	85.6	5817	14.5	
Medium-low	11,335	12.6	9258	81.7	2077	18.3		11,046	13.8	9957	90.1	1089	9.9	
Low	2910	3.2	2384	81.9	526	18.1		2315	2.9	2109	91.1	206	8.9	
**Residence type**							<0.0001							<0.0001
Live with family	85,770	95.3	62,738	73.2	23,032	26.9		77,208	96.1	65,119	84.3	12,089	15.7	
Live with relative	857	1.0	633	73.9	224	26.1		621	0.8	553	89.1	68	11.0	
Accommodation, dormitory	2846	3.2	2203	77.4	643	22.6		2131	2.7	1885	88.5	246	11.5	
Childcare facility	568	0.6	374	65.9	194	34.2		358	0.5	276	77.1	82	22.9	
**Number of days of weight training per week**							<0.0001							<0.0001
More than 5 days a week	14,323	15.9	10,119	70.7	4204	29.4		3513	4.4	2889	82.2	624	17.8	
4 days a week	4766	5.3	3420	71.8	1346	28.2		1539	1.9	1252	81.4	287	18.7	
3 days a week	10,514	11.7	7496	71.3	3018	28.7		4023	5.0	3298	82.0	725	18.0	
2 days a week	13,048	14.5	9529	73.0	3519	27.0		6803	8.5	5615	82.5	1188	17.5	
1 days a week	17,112	19.0	12,596	73.6	4516	26.4		13,859	17.3	11,460	82.7	2399	17.3	
Non-weight training	30,278	33.6	22,788	75.3	7490	24.7		50,581	63.0	43,319	85.6	7262	14.4	
**Time spent sitting and studying on a weekday (hours)**							<0.0001							<0.0001
<8	45,158	50.2	32,410	71.8	12,748	28.2		27,262	33.9	22,703	83.3	4559	16.7	
8 ≤ *n* < 12	19,429	21.6	14,061	72.4	5368	27.6		20,195	25.1	16,902	83.7	3293	16.3	
12 ≤ *n* < 16	15,512	17.2	11,702	75.4	3810	24.6		19,300	24.0	16,371	84.8	2929	15.2	
16 ≤ *n* < 20	9942	11.0	7775	78.2	2167	21.8		13,561	16.9	11,857	87.4	1704	12.6	
**Grade**							<0.0001							<0.0001
High	12,344	13.7	8349	67.6	3995	32.4		8259	10.3	6,430	77.9	1829	22.2	
Medium-high	21,234	23.6	15,322	72.2	5912	27.8		20,132	25.1	16,632	82.6	3500	17.4	
Medium	24,807	27.6	18,022	72.7	6785	27.4		23,508	29.3	19,858	84.5	3650	15.5	
Medium-low	21,020	23.3	15,937	75.8	5083	24.2		20,047	25.0	17,459	87.1	2588	12.9	
Low	10,636	11.8	8318	78.2	2318	21.8		8372	10.4	7,454	89.0	918	11.0	
**Alcohol consumption**							<0.0001							<0.0001
Yes	38,778	43.1	29,950	77.2	8828	22.8		27,254	33.9	24,137	88.6	3117	11.4	
No	51,263	56.9	35,998	70.2	15,265	29.8		53,064	66.1	43,696	82.4	9368	17.7	
**Smoking**							<0.0001							<0.0001
Yes	19,837	22.0	15,544	78.4	4293	21.6		6391	8.0	5732	89.7	659	10.3	
No	70,204	78.0	50,404	71.8	19,800	28.2		73,927	92.0	62,101	84.0	11,826	16.0	
**Depression**							<0.0001							<0.0001
Yes	17,592	19.5	16,102	91.5	1490	8.5		23,307	29.0	22,372	96.0	935	4.0	
No	72,449	80.5	49,846	68.8	22,603	31.2		57,011	71.0	45,461	79.7	11,550	20.3	
**Suicidal ideation**							<0.0001							<0.0001
Yes	8227	9.1	7667	93.2	560	6.8		11,597	14.4	11,236	96.9	361	3.1	
No	81,814	90.9	58,281	71.2	23,533	28.8		68,721	85.6	56,597	82.4	12,124	17.6	
**Total**	90,041	100	65,948	73.2	24,093	26.8		80,318	100	67,833	84.5	12,485	15.5	

**Table 2:** Adjusted association factors associated with physical activity and stress (2015–2017).

Variables	Boys		Girls	
	Adjusted OR	95% CI	Adjusted OR	95% CI
Number of days of intense physical activity per week *				
More than 5 days a week	0.79	(0.78–0.80)	0.77	(0.75–0.79)
4 days a week	0.87	(0.85–0.88)	0.84	(0.82–0.87)
3 days a week	0.89	(0.88–0.91)	0.84	(0.82–0.85)
2 days a week	0.99	(0.98–1.01)	0.90	(0.89–0.92)
1 days a week	1.03	(1.02–1.05)	0.95	(0.93–0.96)
Non-physical activity	1.00		1.00	
**Age**				
14 (middle school 1)	1.00		1.00	
15 (middle school 2)	1.13	(1.12–1.15)	1.13	(1.11–1.15)
16 (middle school 3)	1.15	(1.14–1.17)	1.10	(1.08–1.11)
17 (high school 1)	1.17	(1.15–1.18)	1.32	(1.30–1.34)
18 (high school 2)	1.31	(1.29–1.33)	1.58	(1.55–1.60)
19 (high school 3)	1.22	(1.21–1.24)	1.60	(1.57–1.63)
**Household income**				
High	1.00		1.00	
Medium-high	1.37	(1.36–1.39)	1.46	(1.43–1.49)
Medium	1.53	(1.51–1.55)	1.78	(1.75–1.81)
Medium-low	2.16	(2.13–2.20)	2.23	(2.18–2.28)
Low	1.86	(1.81–1.91)	1.99	(1.91–2.08)
**Residence type**				
Live with family	1.00		1.00	
Live with relative	0.73	(0.70–0.76)	0.90	(0.84–0.96)
Accommodation, trace, dormitory	1.00	(0.98–1.03)	1.04	(1.00–1.08)
Childcare facility	0.42	(0.40–0.44)	0.39	(0.36–0.42)
**Number of days of weight training per week**				
More than 5 days a week	1.00		1.00	
4 days a week	1.04	(1.02–1.06)	1.04	(1.00–1.09)
3 days a week	1.05	(1.03–1.06)	1.10	(1.07–1.14)
2 days a week	1.12	(1.11–1.14)	1.07	(1.03–1.10)
1 days a week	1.12	(1.11–1.14)	1.11	(1.08–1.14)
Non-physical activity	1.20	(1.18–1.21)	1.25	(1.22–1.29)
**Time spent sitting and studying on a weekday (hours)**				
<8	1.00		1.00	
8 ≤ *n* < 12	1.04	(1.03–1.05)	1.04	(1.02–1.05)
12 ≤ *n* < 16	1.22	(1.21–1.24)	1.15	(1.13–1.16)
16 ≤ *n* < 20	1.38	(1.36–1.39)	1.38	(1.36–1.41)
**Grade**				
High	1.00		1.00	
Medium-high	1.14	(1.13–1.16)	1.21	(1.19–1.23)
Medium	1.11	(1.10–1.13)	1.29	(1.26–1.31)
Medium-low	1.24	(1.22–1.26)	1.44	(1.42–1.47)
Low	1.33	(1.30–1.35)	1.53	(1.50–1.57)
**Alcohol consumption**				
Yes	1.17	(1.16–1.18)	1.14	(1.12–1.15)
No	1.00		1.00	
**Smoking**				
Yes	1.03	(1.02–1.04)	0.94	(0.91–0.96)
No	1.00		1.00	
**Depression**				
Yes	3.70	(3.65–3.75)	4.29	(4.21–4.36)
No	1.00		1.00	
**Suicidal ideation**				
Yes	2.74	(2.68–2.80)	3.20	(3.11–3.29)
No	1.00		1.00	

* *p*-value for trend; boys (*p* < 0.0001), girls (*p* < 0.0001). CI: confidence interval; OR: odds ratio.

**Table 3:** Subgroup analysis of the association between intense physical activity and stress stratified by household income, residence type, time spent and studying for a weekday, grade, depression, and suicidal ideation.

Variable	Number of Days of Intense Physical Activity per Week											*p*-Value for Trend
	More than 5 Days a Week		4 Days		3 Days		2 Days		1 Day		Non-Physical Activity	
	OR	95% CI	OR	95% CI	OR	95% CI	OR	95% CI	OR	95% CI	OR	
**Boys**												
**Household income**												
High	0.81	(0.78–0.84)	0.80	(0.76–0.84)	0.91	(0.87–0.95)	1.08	(1.04–1.13)	1.13	(1.09–1.18)	1.00	<0.0001
Medium-high	0.75	(0.73–0.77)	0.88	(0.85–0.90)	0.87	(0.84–0.89)	1.00	(0.97–1.02)	1.00	(0.97–1.03)	1.00	<0.0001
Medium	0.84	(0.82–0.86)	0.88	(0.85–0.90)	0.91	(0.89–0.93)	0.97	(0.95–0.99)	1.02	(1.00–1.04)	1.00	<0.0001
Medium-low	0.76	(0.73–0.79)	0.97	(0.92–1.02)	0.91	(0.87–0.95)	1.05	(1.01–1.09)	1.07	(1.02–1.12)	1.00	<0.0001
Low	0.51	(0.47–0.55)	0.70	(0.63–0.78)	0.83	(0.76–0.91)	0.75	(0.69–0.81)	0.93	(0.86–1.01)	1.00	<0.0001
**Residence type**												<0.0001
Live with family	0.80	(0.79–0.81)	0.89	(0.87–0.90)	0.90	(0.89–0.91)	1.00	(0.99–1.01)	1.04	(1.02–1.05)	1.00	<0.0001
Live with relative	0.49	(0.42–0.57)	0.36	(0.30–0.42)	0.81	(0.69–0.95)	0.89	(0.77–1.05)	1.00	(0.85–1.17)	1.00	<0.0001
Accommodation, trace, dormitory	0.74	(0.67–0.81)	0.72	(0.65–0.81)	0.65	(0.59–0.71)	0.83	(0.76–0.92)	0.95	(0.86–1.05)	1.00	0.0027
Childcare facility	0.49	(0.42–0.58)	0.41	(0.33–0.51)	0.96	(0.79–1.15)	0.63	(0.53–0.76)	0.55	(0.46–0.65)	1.00	0.0366
**Time spent sitting and studying on a weekday (hours)**												
<8	0.78	(0.77–0.80)	0.89	(0.87–0.91)	0.91	(0.89–0.93)	1.00	(0.98–1.02)	1.04	(1.02–1.07)	1.00	<0.0001
8 ≤ *n* < 12	0.79	(0.77–0.81)	0.86	(0.83–0.89)	0.87	(0.85–0.90)	0.98	(0.95–1.01)	1.00	(0.97–1.03)	1.00	<0.0001
12 ≤ *n* < 16	0.76	(0.73–0.78)	0.79	(0.76–0.83)	0.89	(0.86–0.92)	0.97	(0.93–1.00)	0.93	(0.90–0.97)	1.00	<0.0001
16 ≤ *n* < 20	0.84	(0.80–0.88)	0.85	(0.80–0.89)	0.86	(0.82–0.89)	1.03	(0.99–1.08)	1.20	(1.14–1.26)	1.00	<0.0001
**Grade**												
High	0.81	(0.78–0.84)	0.82	(0.79–0.86)	0.85	(0.82–0.88)	1.10	(1.06–1.15)	1.09	(1.05–1.14)	1.00	<0.0001
Medium-high	0.78	(0.76–0.81)	0.90	(0.87–0.93)	0.89	(0.86–0.91)	1.02	(0.99–1.05)	1.13	(1.10–1.17)	1.00	<0.0001
Medium	0.76	(0.74–0.78)	0.88	(0.85–0.91)	0.90	(0.88–0.93)	0.93	(0.90–0.95)	1.01	(0.98–1.04)	1.00	<0.0001
Medium-low	0.83	(0.81–0.86)	0.96	(0.93–1.00)	0.96	(0.94–0.99)	1.00	(0.98–1.03)	1.00	(0.97–1.03)	1.00	<0.0001
Low	0.74	(0.71–0.77)	0.65	(0.61–0.68)	0.83	(0.79–0.87)	0.95	(0.91–0.99)	0.89	(0.86–0.93)	1.00	<0.0001
**Depression**												
Yes	0.59	(0.56–0.63)	0.68	(0.64–0.72)	0.78	(0.74–0.83)	0.85	(0.81–0.90)	0.85	(0.80–0.90)	1.00	<0.0001
No	0.81	(0.80–0.82)	0.88	(0.87–0.90)	0.90	(0.89–0.91)	1.00	(0.99–1.02)	1.04	(1.03–1.06)	1.00	<0.0001
**Suicidal ideation**												
Yes	0.42	(0.39–0.46)	0.54	(0.49–0.60)	0.67	(0.61–0.74)	0.77	(0.71–0.85)	0.86	(0.78–0.95)	1.00	<0.0001
No	0.81	(0.79–0.82)	0.88	(0.86–0.89)	0.90	(0.89–0.91)	1.00	(0.98–1.01)	1.03	(1.02–1.05)	1.00	<0.0001
**Girls**												
**Household income**												
High	0.59	(0.55–0.63)	0.77	(0.72–0.83)	0.81	(0.77–0.85)	0.76	(0.73–0.80)	0.81	(0.77–0.84)	1.00	<0.0001
Medium-high	0.79	(0.76–0.83)	0.92	(0.88–0.96)	0.85	(0.83–0.88)	0.92	(0.90–0.95)	0.98	(0.95–1.00)	1.00	<0.0001
Medium	0.80	(0.77–0.82)	0.86	(0.83–0.90)	0.81	(0.79–0.83)	0.89	(0.87–0.91)	0.93	(0.91–0.95)	1.00	<0.0001
Medium-low	0.92	(0.86–0.99)	0.76	(0.70–0.82)	0.94	(0.89–1.00)	1.08	(1.02–1.13)	1.08	(1.03–1.13)	1.00	0.0011
Low	0.61	(0.53–0.71)	0.37	(0.31–0.43)	0.83	(0.72–0.96)	0.98	(0.87–1.11)	1.10	(0.98–1.23)	1.00	0.0007
**Residence type**												
Live with family	0.80	(0.78–0.82)	0.88	(0.86–0.90)	0.85	(0.84–0.86)	0.90	(0.89–0.92)	0.95	(0.93–0.96)	1.00	<0.0001
Live with relative	0.14	(0.11–0.19)	0.12	(0.09–0.15)	0.48	(0.37–0.62)	1.16	(0.87–1.54)	0.81	(0.64–1.03)	1.00	<0.0001
Accommodation, trace, dormitory	0.65	(0.55–0.76)	0.41	(0.35–0.48)	0.48	(0.42–0.54)	0.91	(0.81–1.02)	1.07	(0.96–1.20)	1.00	0.0006
Childcare facility	0.12	(0.09–0.17)	0.38	(0.27–0.54)	0.62	(0.47–0.81)	0.53	(0.40–0.69)	0.63	(0.48–0.83)	1.00	<0.0001
**Time spent sitting and studying on a weekday (hours)**												
<8	0.76	(0.73–0.79)	0.85	(0.82–0.89)	0.91	(0.88–0.94)	0.92	(0.90–0.95)	0.93	(0.91–0.95)	1.00	<0.0001
8 ≤ *n* < 12	0.93	(0.89–0.97)	0.86	(0.82–0.91)	0.84	(0.81–0.87)	0.93	(0.90–0.95)	1.01	(0.99–1.04)	1.00	<0.0001
12 ≤ *n* < 16	0.71	(0.68–0.74)	0.81	(0.77–0.85)	0.83	(0.81–0.86)	0.84	(0.81–0.86)	0.90	(0.87–0.92)	1.00	<0.0001
16 ≤ *n* < 20	0.63	(0.59–0.67)	0.85	(0.80–0.91)	0.68	(0.65–0.71)	0.94	(0.90–0.97)	0.96	(0.92–0.99)	1.00	<0.0001
**Grade**												
High	0.66	(0.62–0.70)	0.73	(0.68–0.77)	0.75	(0.71–0.78)	0.89	(0.85–0.92)	1.03	(0.99–1.08)	1.00	<0.0001
Medium-high	0.65	(0.63–0.68)	0.87	(0.83–0.91)	0.90	(0.87–0.93)	0.88	(0.86–0.91)	0.92	(0.89–0.94)	1.00	<0.0001
Medium	0.88	(0.84–0.92)	1.03	(0.98–1.08)	0.82	(0.80–0.85)	0.96	(0.93–0.99)	0.90	(0.88–0.93)	1.00	<0.0001
Medium-low	0.82	(0.79–0.86)	0.70	(0.66–0.74)	0.76	(0.74–0.79)	0.82	(0.79–0.85)	0.93	(0.90–0.96)	1.00	<0.0001
Low	0.90	(0.83–0.96)	0.86	(0.78–0.94)	1.09	(1.01–1.17)	1.02	(0.96–1.08)	1.16	(1.10–1.22)	1.00	0.1736
**Depression**												
Yes	0.55	(0.51–0.58)	0.55	(0.52–0.59)	0.70	(0.66–0.74)	0.83	(0.78–0.87)	1.02	(0.97–1.07)	1.00	<0.0001
No	0.81	(0.79–0.83)	0.89	(0.87–0.92)	0.85	(0.84–0.87)	0.91	(0.90–0.93)	0.94	(0.93–0.95)	1.00	<0.0001
**Suicidal ideation**												
Yes	0.57	(0.52–0.63)	0.68	(0.61–0.77)	0.76	(0.69–0.83)	0.93	(0.85–1.01)	1.06	(0.98–1.16)	1.00	<0.0001
No	0.79	(0.77–0.80)	0.86	(0.83–0.88)	0.84	(0.83–0.86)	0.90	(0.89–0.92)	0.94	(0.93–0.96)	1.00	<0.0001
